# Gaps and opportunities in the treatment of relapsed-refractory multiple myeloma: Consensus recommendations of the NCI Multiple Myeloma Steering Committee

**DOI:** 10.1038/s41408-022-00695-5

**Published:** 2022-06-29

**Authors:** Shaji Kumar, Lawrence Baizer, Natalie S. Callander, Sergio A. Giralt, Jens Hillengass, Boris Freidlin, Antje Hoering, Paul G. Richardson, Elena I. Schwartz, Anthony Reiman, Suzanne Lentzsch, Philip L. McCarthy, Sundar Jagannath, Andrew J. Yee, Richard F. Little, Noopur S. Raje

**Affiliations:** 1 Hematologic Malignancies, Mayo Clinic College of Medicine and Science, Rochester, USA; 2 Division of Lung Diseases, National Heart, Lung and Blood Institute, National Institutes of Health, Bethesda, MD USA; 3 Myeloma Clinical Program, University of Wisconsin Carbone Cancer Center, Madison, USA; 4 Division of Hematologic Malignancies, Memorial Sloan Kettering Cancer Center, Madison, USA; 5 Oncology and Internal Medicine, Roswell Park Comprehensive Cancer Center, Buffalo, USA; 6 Biometric Research Program, National Cancer Institute, National Institutes of Health, Bethesda, MD USA; 7 Cancer Research and Biostatistics and University of Washington School of Public Health, Seattle, USA; 8 Jerome Lipper Multiple Myeloma Center, Dana-Farber Cancer Institute, Boston, USA; 9 Coordinating Center for Clinical Trials, National Cancer Institute, National Institutes of Health, Bethesda, MD USA; 10 University of New Brunswick, Department of Medicine, Dalhousie University Department of Oncology, Saint John Regional Hospital, Fredericton, Canada; 11 Multiple Myeloma and Amyloidosis Service, Department of Medicine, Columbia University Medical Center, New York, USA; 12 Department of Medicine, Oncology and Internal Medicine, Transplant & Cellular Therapy Center, Roswell Park Comprehensive Cancer Center, Buffalo, USA; 13 Division of Hematology and Medical Oncology, Mount Sinai School of Medicine, Center of Excellence for Multiple Myeloma, New York, USA; 14 Department of Medicine, Harvard Medical School, Multiple Myeloma Program, Medical Oncology, Massachusetts General Hospital, Boston, USA; 15 Clinical Investigations Branch, Cancer Therapy Evaluation Program, National Cancer Institute, National Institutes of Health, Bethesda, MD USA

**Keywords:** Combination drug therapy, Cancer therapeutic resistance, Targeted therapies

## Abstract

A wide variety of new therapeutic options for Multiple Myeloma (MM) have recently become available, extending progression-free and overall survival for patients in meaningful ways. However, these treatments are not curative, and patients eventually relapse, necessitating decisions on the appropriate choice of treatment(s) for the next phase of the disease. Additionally, an important subset of MM patients will prove to be refractory to the majority of the available treatments, requiring selection of effective therapies from the remaining options. Immunomodulatory agents (IMiDs), proteasome inhibitors, monoclonal antibodies, and alkylating agents are the major classes of MM therapies, with several options in each class. Patients who are refractory to one agent in a class may be responsive to a related compound or to a drug from a different class. However, rules for selection of alternative treatments in these situations are somewhat empirical and later phase clinical trials to inform those choices are ongoing. To address these issues the NCI Multiple Myeloma Steering Committee formed a relapsed/refractory working group to review optimal treatment choices, timing, and sequencing and provide recommendations. Additional issues considered include the role of salvage autologous stem cell transplantation, risk stratification, targeted approaches for genetic subsets of MM, appropriate clinical trial endpoints, and promising investigational agents. This report summarizes the deliberations of the working group and suggests potential avenues of research to improve the precision, timing, and durability of treatments for Myeloma.

## Introduction

The treatment of multiple myeloma has evolved dramatically. The introduction of several new drugs has led to the development of effective combinations, resulting in significant improvement in overall survival [1–4]. Several classes of agents, including proteasome inhibitors (PIs), immunomodulatory agents (IMiDs), alkylators, histone deacetylase inhibitors (HDACi), monoclonal antibodies (MoAbs), antibody drug conjugates (ADC), a novel selective inhibitor of nuclear export (SINE), and, more recently, chimeric antigen receptor T cells, have been approved for treatment of myeloma and used (either alone or in combination) at various stages of the disease [5]. Autologous stem cell transplantation (ASCT) remains a standard of care in younger patients as a consolidation approach in first remission [6, 7]. These drug classes have been used in varying combinations, along with ASCT in transplant eligible patients, to achieve maximal depth of response. Individual drugs or drug combinations are then given continuously as maintenance until disease relapse, with the goal of keeping tumor clones in check [8]. Despite these efforts, myeloma invariably relapses. Evolving initial combination treatment approaches have significantly transformed relapsed disease, with most patients being refractory to one or more drugs at first relapse.

Although novel immunotherapeutic approaches, including cellular therapies and bispecific T cell engagers, have shown great promise, significant challenges remain within the relapsed/refractory space. While a cure remains the ultimate goal, converting myeloma into a chronic disease through sequencing of available therapies judiciously and guided by disease biology, appears to be within grasp for many patients with currently available tools [9, 10]. This paper summarizes discussions within the National Cancer Institute (NCI) myeloma steering committee and a representative group of investigators comprising the relapsed refractory working group. The goal was to identify gaps, challenges, and therapeutic opportunities and identify areas of research likely to lead to improvements in treatment.

## Treatment of patients with relapsed/refractory disease

With current therapies, patients with MM will experience their first relapse at variable intervals after diagnosis [7, 11, 12]. Several important determinants of the subsequent therapy include whether they are on maintenance therapy at the time of relapse and whether they are refractory to maintenance therapies such as lenalidomide, bortezomib or possibly both [9]. It is important to ascertain whether an anti-CD38 monoclonal antibody was part of the initial therapy, or as maintenance or continued treatment (e.g. in the setting of a clinical trial, a likely scenario with the wider adoption of this agent in upfront treatment). Other significant factors include disease-intrinsic characteristics at relapse such as biochemical relapse versus clinical relapse, duration of initial response (with shorter responses identifying high-risk disease), high-risk cytogenetic features, clinical features at time of relapse (e.g. anemia, renal dysfunction, bone lesions), and goals of therapy, including quality of life. Host factors to consider include associated comorbidities, prior toxicities and frailty and its assessment [9, 13].

## Lines of therapy versus resistance to a class of drugs

Single arm studies for new drug development until recently have focused on late-stage patients who had been exposed to all approved agents in more than three prior lines of treatment and were progressing on the last line. In contrast, phase III trials using the new agents in combination involved patients in their first to third lines of treatment. This resulted in an bi-modal approach to treatment: early (first to third) versus late relapse (beyond third). As the number of agents available in myeloma has increased, adoption of these agents in earlier lines of treatment has posed a new challenge to the old paradigm. Therefore, lines of therapy should be modified to include sensitivity to versus resistance to classes of or individual agents. Broadly, the treatment approach to relapsed disease has been that it is preferrable to use triplet regimens, including at least two active drug classes other than steroids, and at least one from a class that the patient has not been exposed. If a class is repeated, a different drug from the class should be used. This approach can be employed in earlier relapses with drugs that the patient has not been exposed to but can be more difficult to implement in later relapses. Studies have shown that drugs can be effectively reused, especially when the patient has not been exposed to them for a while, thus allowing new combinations in later stages [14, 15]. The general approach has been to continue at least one drug in the combination as maintenance (if tolerable) until disease progression.

## First relapse in patients with lenalidomide-refractory disease

Current practice, especially in the US and Canada, with the use of lenalidomide in all upfront regimens and the adoption of lenalidomide maintenance or continuous use results in most patients being lenalidomide refractory at first relapse. This growing population requires study, with an additional consideration that lenalidomide resistance can vary in that a patient who has progressed on full dose lenalidomide in combination with other agents is likely different from a patient who progresses on single agent low dose lenalidomide. Although all pivotal trials have demonstrated the benefits of triplet combinations, patients with lenalidomide-refractory disease were excluded from recent randomized phase 3 trials testing Rd (lenaldomide/dexamethasone) versus Rd plus a third agent (either a PI, [carfilzomib, KRd [16], or ixazomib, IRd [17, 18]] or a MoAb, [elotuzumab, Elo-Rd [19], or daratumumab, DRd] [20]).

Relevant to this issue are several Phase 3 trials that evaluated PI-based combinations using Vd as the control arm in RRMM. For example, in the Phase 3 OPTIMISMM trial the combination of pomalidomide plus Vd (PomVd) was prospectively compared to Vd in patients with RRMM who had received one to three prior lines of therapy that included lenlidomide [21]. More than 70% of the patients were refractory to lenalidomide. After a median follow-up of 16 months, PomVd demonstrated an improved median PFS (11.2 versus 7.1 months; HR 0.61; *P* < 0.0001). The median PFS was also prolonged with PomVd in patients refractory to lenalidomide (9.5 versus 5.6 months, *P* = 0.0008) and in patients with one previous line of treatment (20.7 versus 11.6 months, *P* = 0.0027). Of particular interest were the results in patients who had received one previous line of treatment and were refractory to lenalidomide (17.8 versus 9.5 months, *P* = 0.03). In addition, both carfilzomib and ixazomib have been combined with pomalidomide and dexamethasone in phase 2 trials and provide alternative options when a combination of a PI and an IMiD is desired (e.g. the Alliance A061202 trial) [22, 23]. Further details on these trials are included in Table 1.

**Table 1:** Results of randomized clinical trials in Lenalidomide-refractory patients.

Treatments for first relapse in patients with lenalidomide-refractory disease				
Trial	Phase	Agents	Outcome	References
OPTIMISMM	III	POM-Vd vs. Vd	Median PFS 11.2 vs. 7.1 months	[21]
A061202	II	POM-Kd	Median PFS 7.2 months Median OS 20.6 months	[23]
	I/II	POM-Kd	Median PFS 10.3 months 1 year OS 67%	[22]
CANDOR	III	Dara-Kd vs. Kd	Median PFS not reached vs. 15.8 months	[24]
IKEMA	III	Isa-Kd vs. Kd	Median PFS not reached vs. 19.1 months	[25]

*POM* pomalidomide, *V* velcade, bortezomib, *d* dexamethasone, *Dara* daratumumab, *K* carfilzomib, *Isa* Isatuximab.

Combinations of Kd and anti-CD38 antibodies were recently evaluated in phase 3 studies. In the CANDOR trial, Kd was prospectively compared with Kd + daratumumab (Dara-Kd) in RRMM patients who had received one to three prior lines of therapy (446 patients, 33% lenalidomide refractory) [24]. This study showed that the median PFS was not reached for the Dara-Kd group, and was 15.8 months for the Kd group (HR 0.63, *P* = 0.0027). Similarly, in the phase 3 IKEMA trial (reported at EHA 2020 meeting), 302 patients with RRMM and 1–3 prior lines of therapy were randomized to receive either Isatuximab plus Kd (Isa-Kd, *n* = 179) or Kd (*n* = 123) [25]. At a median follow-up of 20.7 months, median PFS was not reached for Isa-Kd versus 19.1 months for Kd (HR 0.53; *P* = 0.0007). Isa-Kd was superior to Kd in terms of PFS for both lenalidomide-exposed (HR 0.50) and lenalidomide-refractory patients (HR 0.60). These combinations may be important options for first relapse in patients with lenalidomide-refractory disease, without recent carfilzomib exposure.

Anti-CD38 antibodies have also been combined with pomalidomide in phase 3 trials that included a subgroup of patients refractory to lenalidomide. In the APOLLO trial, daratumumab added to Pom-Dex led to improved PFS for the triplet and in the ICARIA trial, isatuximab added to Pom-Dex led to improved PFS and OS compared with Pom-Dex [26, 27]. Additional studies in lenalidomide-exposed patients evaluated Vd+/− venetoclax [28] or Vd+/− Selinexor [29]. These studies have only a subset of patients who are refractory to lenalidomide.

The Spanish group conducted a phase 2 trial comparing carfilzomib with cyclophosphamide and dexamethasone to carfilzomib and dexamethasone, demonstrating improved PFS for the triplet. The study specifically examined the outcomes among lenalidomide refractory patients, demonstrating a significant improvement for KCd.

The approval of daratumumab-based regimens as first line therapy in myeloma (DaraVMP [30], Dara-Rd [12], and Dara-VTD [11] and Dara-RVd [31], although the last combination is not yet FDA approved) makes decisions regarding second line treatment challenging. There is no prospective data yet for daratumumab retreatment at second line (although a retrospective analysis suggested possible benefit from Dara re-treatment [32]), and salvage therapy using isatuximab in patients progressing on daratumumab is an unlikely option. Combinations such as KD, VCD, PVD, KCd, or KPD are reasonable options; alternatively, VD-Elo, SVD, or IPD could be considered.

## Bortezomib refractory patients in first relapse

Patients relapsing after receiving bortezomib containing regimens were well represented in the POLLUX trial, comparing DRd versus Rd [20]; at least 85% of patients were proteasome inhibitor exposed and both PI refractory and exposed responded equally well to the DRd combination, with long-term follow-up showing a PFS of 44.5 versus 17.5 months for Rd [33]. The phase 3 ENDEAVOR trial compared bortezomib retreatment using a doublet (with dexamethasone) with carfilzomib and dexamethasone and showed a PFS benefit favoring Kd of 18.7 vesus 9.4 months, with a modest OS benefit for 47.8 versus 38.8 months [34]. However, the frequency of side effects with this intensive schedule and the preference for triple therapy limit the applicability of this approach [35]. Previously mentioned regimens such a EloPd, DPd, Isa-Pd, KCd, or KRd would be reasonable choices in bortezomib refractory patients relapsing in the first line, although some are not yet FDA-approved for second line treatment.

## Salvage ASCT

Frontline ASCT remains the standard of care for fit patients in many countries, with some age limits [6, 7]. Nevertheless, absent an OS benefit of front-line ASCT in patients with standard risk disease when compared to RVD followed by lenalidomide maintenance, for example, some investigators and patients prefer to delay ASCT until first relapse, after harvesting and storing stem cells during induction [36, 37]. In this setting, salvage ASCT should be systematically considered in patients who have not received a transplant [38–40]. One issue is the selection of the optimal re-induction regimen prior to salvage ASCT given upfront as a long-term therapy, especially for patients progressing on lenalidomide. Minimal data is available regarding re-induction regimens. KPD was found to be active in this setting in a phase 2 study conducted by the HOVON group and PCd is an additional option [41].

The role of a second ASCT has been explored in two randomized trials, with the majority of evidence coming from single institution or registry studies. Cook et al. randomized 297 patients to receive high dose melphalan and a salvage auto HCT (*n* = 89) or continued oral cyclophosphamide after reinduction therapy (*n* = 85). Updated time to disease progression results show a significant advantage in the salvage ASCT group compared to weekly cyclophosphamide (19 versus 11 months; HR 0.45 [95% CI 0.31–0.64] *p* < 0.0001). Median overall survival was also superior in the salvage ASCT group (67 versus 52 months; HR 0.56 [0.35–0.90], *p* = 0.0169) [42]. The German ReLApsE trial randomized patients in 1st–3rd relapse post ASCT to receive a 2nd ASCT or continued Rd therapy. Intent to treat analysis, indicated that there were no differences in ORR between the two arms (75% versus 78%). However, 1 of 3 patients randomized to the ASCT arm did not receive the assigned therapy. A landmark analysis at the 5th Rd cycle in arm A and at ASCT in arm B showed a trend for improved PFS with ASCT as well as improved OS [38, 43, 44].

The CIBMTR recently published the largest registry experience with 975 patients undergoing salvage ASCT between 2010 and 2015. The 3-year PFS and OS outcomes were 13% and 68%, respectively. Patients who relapsed ≥36 months after first ASCT had significantly better PFS and OS than those relapsing earlier (3-year PFS, 16% versus 9%; *p* = 0.01); (3-year OS, 72% versus 61%; *p* = 0.004), respectively [45].

In the context of modern treatments, the impact of salvage ASCT has come into question. Two studies have shown increasing depth of response with salvage ASCT after reinduction with modern triplet or quadruplet therapy. Shah et al. presented the interim analysis of a phase II trial evaluating the efficacy of daratumumab, carfilzomib, lenalidomide, and dexamethasone (Dara-KRD) with high-dose melphalan and autoHCT in patients with 1–3 prior lines of therapy. Patients received 4 cycles of Dara-KRD followed by ASCT and 4 additional cycles of Dara-KRD followed by maintenance. Twenty-three patients enrolled with 22 evaluable for interim analysis. 86% of patients were in 1st relapse before enrollment. 82% underwent ASCT and 59% completed all study treatments. Best response was a CR in 45%, >VGPR in 77%, and >PR in 82% [46].

Baertsch et al. reported similar results in 44 patients receiving salvage ASCT following re-induction with KRd. After reinduction and transplant 77% of patients achieved a VGPR or better. Median PFS was 23.3 months post salvage transplant. Patients with ≥VGPR to salvage ASCT and those receiving maintenance treatment post-transplant salvage had superior PFS (HR 0.19 and OS HR 0.20). These results need to be prospectively compared to other established and emerging therapies [47].

The American Society of Blood and Marrow Transplantation (ASBMT), together with the European Bone Marrow Transplant Society and International Myeloma Foundation published guidelines supporting the use of 2nd transplants in patients whose remissions initial remission lasted 18–24 months. Prospective trials will be essential to document the benefit of this approach in patients with RRMM, particularly those who have received new classes of agents. In the era of greater success with CAR-T cell therapy and bispecific antibodies in relapsed and refractory myeloma, the role of autologous stem cell transplant is better reserved upfront. Availability of stem cells allows patients to be rescued from cytopenia due to advanced disease, extensive prior therapies or delayed cytopenia from CAR-T cell therapy. Trials of salvage autologous transplantation are summarized in Table 2.

**Table 2:** Outcomes of trials of salvage ASCT.

Salvage autologous stem cell transplantation in relapsed/refractory MM patients				
Trial	Phase	Agents	Outcome	References
BSBMT/UKMF Myeloma X	III	High dose Melphalan + ASCT vs. oral cyclophosphamide	Time to progression 19 vs. 11 months; OS 67 vs. 52 months	[42]
ReLApsE	III	Second ASCT vs. continued Rd	No difference in response rates; trends toward improved PFS and OS with ASCT	[38, 43, 44]
2nd Chance	II	4 X Dara-KRD→ASCT →4 X Dara-KRD →Maintenance	CR 45%, >VGPR 77% >PR 82%	[46]
Retrospective analysis	II	KRd→ASCT	≥VGPR 77%, median PFS 23.3 months	[47]

*ASCT* autologous stem cell transplant, *Rd* Lenalidomide-dexamethasome, *Dara-KRD* Daratumumab-Carfilzomib Lenalidomide dexamethasone, *KRD* Carfilzomib Lenalidomide dexamethasone.

## Treatment of patients with relapsed or refractory disease who have received two or more prior lines of therapy

The treatment of patients who have received two or more prior lines of therapy is becoming particularly challenging. Lenalidomide and bortezomib are common as part of frontline therapy or at first relapse. Monoclonal antibodies and carfilzomib are also increasingly used during the first two lines of treatment. Therefore, upon second relapse, all agents listed for use at first relapse that have not been tried so far, or for which the patient has not been shown to be refractory, can be used (e.g., bortezomib used as induction treatment before ASCT but not thereafter). A clinical trial, when available, should always be considered.

Baz et al. compared the efficacy of pomalidomide-cyclophosphamide-dexamethasone (PomCyDex) and pomalidomide-dexamethasone (PomDex) in patients who had received four prior lines of therapy. Progression-free survival was increased from 4.4 to 9.5 months with the addition of cyclophosphamide [48].

Selinexor, a SINE compound that blocks exportin 1 and forces nuclear accumulation and activation of tumor-suppressor proteins, has been evaluated in combination with dexamethasone in patients previously exposed to bortezomib, carfilzomib, lenalidomide, pomalidomide, daratumumab, and an alkylating agent, with disease refractory to at least one PI, one IMiD, and daratumumab (triple-class refractory) in the phase 2 STORM study [49]. A partial response or better was observed in 26% of the 122 patients (53% of whom had high-risk cytogenetic abnormalities). The median PFS was 3.7 months, and the median OS was 8.6 months. A pre-specified subgroup analysis of 83 patients with disease refractory to bortezomib, carfilzomib, lenalidomide, pomalidomide, and daratumumab showed an ORR of 25.3%; the median response duration was 3.8 months. Based on this, the FDA accelerated approval to selinexor for treatment of this subgroup of patients in July 2019. One issue with this oral agent is the safety profile; one-fourth of the patients experienced grade 3 fatigue, gastrointestinal toxicity, and thrombocytopenia. The phase 3 BOSTON study evaluated weekly Selinexor, dexamethasone, and bortezomib or dexamethasone and bortezomib twice weekly in patients who had previously been treated with one to three lines of therapy, including proteasome inhibitors. Median progression-free survival was 13.9 months (95% CI 11.7-not evaluable) with selinexor, bortezomib, and dexamethasone and 9.5 months (8.1–10.8) with bortezomib and dexamethasone. Peripheral neuropathy of grade 2 or higher was less common in the Selinexor group [29]. However, as mentioned, GI and other toxicities of Selinexor can be significant, and other SINE compounds are being evaluated to discover agents that may be better tolerated [50].

B-cell maturation antigen (BCMA) promotes MM pathogenesis in the bone marrow microenvironment and is a specific MM target antigen. Belantamab mafodotin is anti-BCMA ADC auristatin immunotoxin and is a first-in-class anti-BCMA treatment recently approved by the FDA. In the DREAMM-2 phase 2 study, 196 patients with triple-class refractory MM received two different doses of Belantamab mafodotin (2.5 mg/kg [*n* = 97] or 3.4 mg/kg [*n* = 99]) [51]. Overall response was 31% and 34% for the two doses, respectively. The median PFS was 2.9 months in the 2.5 mg/kg cohort and 4.9 months in the 3.4 mg/kg cohort (OS data were not mature at the time of publication). The most common grade 3–4 adverse events included keratopathy (27% and 21% of patients for the two doses, respectively), thrombocytopenia and anemia. Based on these data, belantamab mafodotin was approved for RR MM treatment. Inclusion of this agent in the FDA-supported Risk Evaluation and Mitigation Strategy (REMS) program allows tracking and mitigation of keratopathy-related eye problems.

Finally, in the ELOQUENT 3 study of patients who are refractory or relapsed and refractory to lenalidomide and a proteasome inhibitor, the risk of progression or death was significantly lower in those treated with elotuzumab plus pomalidomide and dexamethasone [33, 52]. Details of these trials are included in Table 3.

**Table 3:** Possible treatments for patients who have received two or more prior lines of therapy.

Treatment of patients who have received two or more prior lines of therapy				
Trial	No Prior Lines	Agents	Outcome	References
BOSTON	1–3	Weekly Selinexor-Vd vs. 2 X per week Vd	Median PFS 13.9 months vs. 9.5 months. Less PN with Selinexor-Vd	[29]
APOLLO	2	Dara-POM-d vs. POM-d	Median PFS 12.4 vs. 6.9 months	[27]
ICARIA	3	Isa-POMd vs. POMd	Median PFS 11.5 vs. 6.5 months	[26]
DREAMM-2	3	Belantamab mafadotin 2.5 or 3.4 mg/kg	ORR 31% and 34% Median PFS 2.9 and 4.9 months	[51]
ELOQUENT 3	3	Elotuzumab-POMd vs. POMd	ORR 53% vs. 26% Median PFS 10.3 vs. 4.7 months	[52]
	4	PomCyDex vs. PomDex	Median PFS 9.5 vs. 4.4 months	[48]
STORM	7	Selinexor + dexamethasone	≥PR 26%, median PFS 3.7 months Median OS 8.6 months	[49]

*Vd* bortezomib + dexamethasone, *POMd* pomalidomide + dexamethasone.

## Promising investigational options

### Other BCMA-targeted therapies

Immunologically-based therapies targeting BCMA demonstrate promise independent of genetic heterogeneity and genetic risk, even in MM patients without other treatment options. The continued expression of BCMA in some patients relapsing on BCMA-based therapies provides the option of continuing to target this antigen. Agents include antibody-drug conjugates (ADCs), autologous chimeric antigen receptor engineered T cells (CAR-T), and bispecific T cell engagers [53, 54].

Initial trials of CAR-T cell therapy showed encouraging results in MM. In a phase 1 dose escalation/expansion study of idecabtagene vicleucel (ide-cel, bb2121), a BCMA-targeting CAR T-cell construct, 33 of 36 enrolled patients received CAR-T cells after lymphodepleting chemotherapy. Toxicities were manageable and response rates were high, including a 45% CR rate. In view of these promising results, the KarMMa Phase 2 trial evaluated the efficacy and safety of bb2121 in 128 patients with a median of 6 prior lines of therapy, 94% refractory to anti-CD38 monoclonal antibody therapy. Responses were observed in 73% of patients, with a 33% CR rate. High rates of MRD negativity, manageable toxicities, and promising progression-free and overall survival led to FDA approval of bb2121 for the treatment of adult patients with relapsed or refractory multiple myeloma after 4 or more prior therapies [55]. Ciltacabtagene autoleucel (cilta-cel) is closely following ide-cel in clinical development with impressive efficacy. In a similar relapsed/refractory patient population as ide-cel, the phase I/II CARTITUDE-1 trial evaluated 97 patients with a median of 6 prior lines of therapy, 88% triple class refractory. The study demonstrated an ORR of 96.9% with 34% MRD-negative complete response or stringent complete response. Recently updated results showed a 2-year PFS of 60.5% [56].

Bispecific antibodies bind to CD3 on T-cells and BCMA on myeloma cells, activating T-cells and lysing targeted cells. There are several programs in active development including, CC-9329, elranatamab, REGN5458, TNB-383B and teclistamab. Results from the phase 1/2 study of teclistamab in triple-class exposed, relapsed disease (*N* = 150) showed promising outcomes, with an ORR of 62% and 9-month PFS of 58.5% [57, 58]. GPRC5D is an orphan G protein-coupled receptor expressed at significantly higher levels on myeloma cells compared with normal plasma cells targeted by the talquetamab bispecific antibody, which has shown promise in early studies [59]. Fc receptor-homolog 5 (FcRH5) is a type I membrane protein expressed on B cells and plasma cells, and is found on myeloma cells with near 100% prevalence. FcRH5 is targeted by the BCFR4350A BITE, which has also shown some initial promise [60].

### BCL-2 inhibition

Venetoclax, a selective BCL-2 inhibitor, was initially investigated as in combination with carfilzomib and dexamethasone in RRMM patients harboring a t(11;14) and yielded a response rate of 80% in all patients, 92% in those with the translocation. CRR or better was seen in 41% of patients and median progression-free survival was 22.8 months [61]. Venetoclax was subsequently investigated in combination with Vd in the BELLINI phase 3 study. While progression free survival significantly improved with the addition of venetoclax, early deaths increased with this agent. Interestingly, a significant PFS benefit was reported with Vd-venetoclax among patients with t(11;14) (HR = 0.10, 95% CI: 0.02–0.46, *P* = 0.003) and high bcl-2 expression (HR = 0.26, 95% CI: 0.13–0.51, *P* < 0.001). Vd-venetoclax was also superior to Vd in terms of PFS (HR = 0.26, 95% CI: 0.14–0.48, *P* < 0.001) and MRD negativity rate (19% versus 0) for the combined group of patients with t(11;14) or high bcl-2 expression. For this subgroup of patients with a longer follow-up, Venetoclax did not adversely impact OS [28]. In the ongoing CANOVA phase 3 trial (NCT03539744), venetoclax plus dexamethasone is being compared to pomalidomide-dexamethasone in patients with RRMM with t(11;14) refractory to lenalidomide. This agent, not yet approved, may become the first targeted therapy for RRMM with t(11;14) and/or high bcl-2 expression [62, 63]. Trials of investigational agents are described in Table 4.

**Table 4:** Trials of investigational agents in relapsed/refractory myeloma.

Investigational agents: Immunological approaches				
Trial	Phase	Agents	Outcome	References
KarMMa	II	idecabtagene vicleucel ide-cel, bb2121	ORR 73%, CR 33%, 26% MRD-, median PFS 8.8 months	[53]
CARTITUDE-1	I/II	Ciltacabtagene autoleucel (cilta-cel)	ORR 96.9%, 34% MRD-negative CR, 2-year PFS 60.5%	[54]
MajesTEC-1	I/II	teclistimab	ORR 62%, 9-month PFS 58.5%	[55, 56]
**Investigational agents: BCL-2 inhibition**				
	II	Venetoclax-Kd	ORR 80% in all patients, 92% in t(11:14). ≥CRR 41% median PFS 22.8 months	[59]
BELLINI	III	Venetoclax-Vd vs. Vd	Median PFS 22.4 vs. 11.5 months, increased overall mortality with Venetoclax. Venetoclax-Vd increased PFS in patients with t(11;14) (HR = 0.10) or high bcl-2 expression (HR = 0.26), also increased MRD- rate (19% vs. 0) with no increased mortality	[60]

*Kd* carfilzomib-dexamethasone, *Vd* bortezomib-dexamethasone.

## Gaps in RR MM

Despite the availability of these options, important questions remain unanswered. During the past 10–20 years, large Phase 3 trials have been focused on drug approval and have failed to answer several important questions. The following sections outline questions and priorities discussed within the group.

## Optimal timing of restarting therapy

The disease course can be heterogeneous in patients with multiple myeloma, ranging from aggressive relapse with hypercalcemia, new lytic bone lesions, extramedullary disease, or plasma cell leukemia at one extreme to a slow increase in the monoclonal protein over a prolonged period with no evidence of end organ damage at the other [9, 13]. Given this diversity, there is significant variation in the standard practice and approach of therapy initiation for relapsed myeloma, with some patients being initiated on therapy with any increase in monoclonal protein and others not starting therapy until clear evidence of end organ damage due to disease. While this may appear to be similar to the management strategy for patients with smoldering multiple myeloma, relapsed patients have already demonstrated symptomatic disease requiring therapy and hence may have a different disease course than typically observed in the precursor phase [64–66]. Although early intervention in smoldering myeloma has shown benefit in terms of progression free survival, there is currently no prospective data to guide treatment for the relapsed patient [67–69]. In routine practice, many physicians would initiate therapy at the earliest evidence of progression in patients with high-risk disease as well as in those patients initially presenting with significant end organ damage like neurological complications from bone involvement or renal failure. There are many patients, especially after stem cell transplant, who have either become immunofixation positive after achieving a complete response or have low and slowly increasing levels of monoclonal protein or light chains. Clinical trials should be designed to examine the impact of early intervention in all patients with relapsed myeloma to identify the appropriate timing of intervention when organ manifestation is absent, particularly in patients whose laboratory criteria do not meet IMWG definitions of progression and to determine the appropriate intensity of therapy in relation to disease characteristics such as cytogenetic risk.

## Risk stratification in relapsed disease

Given the heterogeneity of the clinical course in multiple myeloma, there has been significant interest in developing risk stratification systems to guide prognostication and therapeutic decisions. Risk models relevant to newly diagnosed MM, such as the Revised International Staging System (R-ISS), have become widely accepted and are uniformly evaluated at the time of diagnosis. However, although many prognostic factors have been identified in relapsed myeloma, there has not been any formal risk stratification system proposed or accepted in the relapsed setting [68]. Studies show that many risk factors identified for newly diagnosed myeloma can inform the setting of relapsed disease, including the R-ISS System, patient frailty, acquisition of new genetic abnormalities such as 1q amplification, extramedullary disease, and circulating plasma cells [70–74]. At the time of relapse there would be additional risk factors that became evident since diagnosis, particularly the duration and depth of response to initial therapy. It has become clear that patients with short lasting responses or primary refractory to initial therapy have very poor outcomes and need to be considered as having high-risk disease. As with newly diagnosed disease in the past, current treatment of relapsed myeloma is relatively homogeneous. Lessons from the setting of newly diagnosed disease indicate that therapy for relapsed disease should be based on risk assessment, requiring the formal development of a risk stratification system that can be uniformly applied in clinical practice and across clinical trials; this will allow unique approaches for relapsed disease based on underlying disease biology and host characteristics.

## Selection of optimal regimen

Significant progress has been made in therapies for multiple myeloma, both in upfront therapy and relapsed disease, which has resulted in improved survival. Although myeloma does not appear curable with current treatment strategies, and is characterized by repeated relapses, delivering the best possible therapy with the least toxicity at each relapse will likely result in the best long-term outcomes. Several large Phase 3 trials focused on evaluating the efficacy of new drugs used in combination with existing drugs have been conducted over the past decade. Many of these trials have repeatedly shown that a three-drug combination improves both progression free and overall survival, suggesting a multidrug combination that likely impacts the clonal diversity of the tumor will provide the best clinical results [75]. In the past, when the drug classes available for the treatment of myeloma were limited, treatment decisions were uncomplicated. However, with the increasing availability of new drug classes and multiple drugs within each class, a nuanced, data-based decision-making process is needed. Randomized trials evaluating treatment approaches, stratified for numbers of relapse and disease heterogeneities (including time of relapse, disease burden, genetic evolution, clinical presentation, associated co-morbidities and treatment history) will help identify treatment strategies with the optimal long-term outcome.

## Response adapted therapeutic strategies, including discontinuation

The depth of response to any therapy often determines efficacy in controlling the disease, a phenomenon seen both in newly diagnosed and relapsed myeloma [74]. While even achieving stable disease has been shown to have clinical benefits in late-stage relapse, in the earlier relapses it would be appropriate to achieve the optimal response while balancing the potential toxicity of treatment regimens. However, limited data exist in relapsed disease to indicate whether basing therapy on response depth can alter long-term outcomes. This important question needs to be answered in relapsed disease, as it has been for newly diagnosed myeloma. In this context, the role of minimal residual disease negativity in the relapse setting is not well defined. Recent Phase 3 trials have repeatedly shown that patients who achieve a minimal residual disease negative status have a better progression free survival, and sometimes better overall survival as well. However, it remains unclear whether achieving MRD negativity only defines treatment sensitive myeloma or if MRD status is a reliable indicator of therapeutic effectiveness and for patients with detectable MRD, to direct for further therapeutic strategies, including intensification of therapy in MRD positive patients or discontinuation/de-escalation of therapy in those who achieve MRD negativity. The latter is particularly important, as many of the industry-sponsored phase 3 trials in the relapsed setting have involved continued therapy until disease progression. This approach, although possibly suitable for some patients with myeloma, is unlikely to be required for everyone and can significantly increase toxicity and health care costs. As a result, there is an urgent need to define the ideal duration of therapy in the relapsed setting and further refine the nature of regimens that can be used for continued therapy or maintenance. CAR-T cell therapy can provide a high CR/MRD negative response rate without maintenance, but response durations can be variable. Accordingly, much effort is currently being directed to increasing persistence of CAR-T cells in myeloma [76, 77]. Achievement of that goal will permit studies to address questions related to response-adapted treatments in the future.

## Targeted approaches for specific genetic subsets

Multiple myeloma is a heterogeneous disease from a genetic standpoint, and a multitude of genetic abnormalities have been described. These include structural chromosomal abnormalities including translocations, karyotypic abnormalities that include monosomies and trisomies, mutations that appear to accumulate over time and changes in copy number [78, 79]. Most of the effective drugs in current use to treat myeloma are not strictly targeted agents but work through pathways common to all plasma cells. However, there is clear proof of principle that many genetic abnormalities can be targeted using specific drugs, which could potentially improve outcomes in these patient subgroups. One example has been the use of venetoclax in patients with translocation (11;14), where the drug used in combination with dexamethasone, can lead to responses in over 2/3 of patients previously refractory to available therapies [28, 63]. Specific mutations can also be targeted, as shown in the setting of relapsed disease carrying mutations involving NRAS, KRAS, and B-RAF [79–81]. Umbrella trials that allow the evaluation of molecularly targeted drugs and drug combinations in patient subgroups with the corresponding genetic abnormalities may improve current therapeutic approaches. In this context, it will be important to better define both selection criteria and assessment of response in these patients, given the sub clonal nature of many of these mutations. Similarly, approaches such as high throughput drug screening should be investigated to repurpose existing drugs to treat relapsed multiple myeloma.

## Optimizing the role of immunotherapies and combinations

Immune-based therapeutic approaches represent the next wave of progress in myeloma. Recent trials demonstrating the power of immunotherapy in myeloma have incorporated monoclonal antibodies with or without drug conjugates, chimeric antigen receptor T (CAR T) cell therapy, and bispecific antibodies that enhance T cell responses to the tumor. These drugs have clearly improved outcomes in patients who have become refractory to the available drugs. Unfortunately, some responses have not been durable, raising the question of whether these therapies should be initiated earlier, including at first relapse and possibly as the initial therapy. Questions remain regarding immunotherapies, including the optimal timing for specific therapeutic platforms with respect to disease burden at therapy initiation, specific combinations that can enhance efficacy, post-therapy interventions that can extend the durability of response with one-time treatment therapies like CAR T cells and understanding the role of the immune micro-environment. In particular, sequencing of therapies that target B cell maturation agent (BCMA) merits exploration, as do the mechanisms that lead to therapeutic failure, including deletion of the BCMA gene [82, 83], increased levels of soluble BCMA and whether treatment with a different BCMA directed therapy after failure of the first attempt produces meaningful responses [84].

## Trials addressing sequencing and retreatment

The current approach to sequencing different therapeutic regimens in patients with multiple relapses has been based primarily on the drugs to which patients have become refractory. Conventional wisdom has been to use triplet therapies when possible based on multiple Phase 3 trials that have demonstrated an overall survival improvement with triplets compared to doublet therapies. In addition, most practitioners would consider introducing at least one new class of drugs and, if possible, two new classes in combination with dexamethasone. Important questions concerning sequencing include whether it would be best to change both classes of drugs, and if changing one drug type to another from the same class would give similar long-term outcomes. As myeloma continues to be a chronic disease with multiple relapses, the question of re-treatment with drugs the patients have previously been exposed to will arise. Few clinical trials have examined the benefit of retreating a patient with a drug to which they previously stopped responding. Available data suggest the longer the interval from the prior exposure, the more likely a response, but there are limited data on the durability of responses to re-treatment.

## Endpoints for clinical trials in relapsed disease

Deciding on appropriate clinical trial endpoints must take into account the phase of the trial, the question(s) being addressed, and the overall goal of the trial. Most clinical trials in the relapse setting have relied on traditional endpoints such as overall survival (Phase 3), progression free survival (Phases 2 and 3), event-free survival (Phases 2 and 3), response rates (Phase 2), and toxicity/feasibility (Phase 1 and pilot studies). Choice of endpoint also depends on whether a trial is being conducted to examine an induction or maintenance question and whether there are regulatory goals in mind. If the trial is intended to inform the principles and practice of medicine, then endpoints reflecting true clinical benefit are required. However, the increasing pace of introduction of new therapeutics could pressure trials intended for regulatory approvals to focus on early endpoints. This may be justified for new drug approvals because the efficacy of current therapies has improved survival substantially. Accordingly, an overall survival endpoint requires larger trials with longer times to completion.

Consequently, interest in surrogate endpoints predictive for true clinical benefit has intensified. It should be emphasized that to be clinically relevant, a surrogate endpoint must be a proper trial-level surrogate that has been validated for a specific type of therapy (e.g., a class of drugs with a certain mechanism of action) in a specific clinical setting (e.g., induction versus maintenance) and in a specific salvage-therapy context. Other endpoints such as PFS2, time to approach failure (TAF), and time to treatment failure (TTF) have been considered acceptable for establishing meaningful clinical benefit in some settings [85–88]. Regulatory authorities have also developed pathways for provisional drug approvals based on early endpoints as randomized clinical trials with clinically meaningful endpoints are completed for final and full new drug approval. As durations of treatment responses continue to improve, even progression free survival may become less feasible as an endpoint in terms of developing and introducing new therapies for myeloma. Therefore, ongoing efforts are directed at evaluating minimal residual disease (MRD) negativity as a surrogate endpoint in clinical trials. MRD negativity, although shown to be a good prognosticator of survival (especially progression free survival) may have to be combined with other measurements, especially in the relapsed refractory disease setting. This may include assessment of the immune repertoire in the context of excellent disease control and imaging to rule out extramedullary disease. In addition, quality of life assessment will become an increasingly important part of the overall assessment of a given drug’s benefit and will likely play an important role as an endpoint. Recent studies suggest that with targeted therapies there may be selective elimination of certain tumor clones, which may not result in comparable improvement in conventional disease parameters such as monoclonal protein levels. Thus, changes in clonal diversity with targeted therapy and their relation to conventional outcomes such as progression free survival and overall survival need to be understood. Surrogacy validation and interpretation of the clinical implications of biomarkers requires these efforts be embedded in well-designed studies with adequately powered true clinical benefit (survival) endpoints. As the industry pursues provisional drug approvals based on early endpoints, there is an opportunity for the publicly funded NCTN myeloma groups to define the clinical benefit of new therapies and combination therapies and to define the biomarker science that will guide future drug development and clinical decision making.

## Definition of lines of therapy versus class refractoriness

The efficacy of any given therapy in myeloma is heavily dependent on prior exposure to other myeloma drugs, especially those in the same class. In addition, the status of refractoriness to a given drug, the number of different regimens the patient has been exposed to and the time during which these different regimens have lost efficacy influences the effectiveness of therapy. To be able to compare different clinical trials in the relapsed setting patients have to be defined in a uniform fashion with respect to their prior therapies. Current practice considers lines of therapy when comparing different clinical trial populations and in stratifying patients in randomized clinical trials [89, 90]. However, this practice assumes a uniform line of therapy definition. Diverse drug choices and different permutations and combinations makes it increasingly difficult to understand the impact of the number of lines of therapies patients have received. Additional issues include gaps in therapy due to toxicity or patient intolerance which are not necessarily considered when describing lines of therapy; dose reductions are another factor. It is likely a new paradigm based on drug classes to which a patient’s disease is refractory, rather than the number of lines of therapy, is necessary. However, this requires analysis of large datasets in a retrospective fashion and eventual prospective validation.

## Trial designs for demonstrating the efficacy of combinations

The traditional approach to assess efficacy of newer therapies in Phase 3 settings has been to add a new drug to a standard of care and compare the two to demonstrate effectiveness. This becomes increasingly difficult as triplets and quadruplets grow more common as treatments. It is difficult to combine more than four or five drugs at a time, both from toxicity and cost standpoints. It is imperative to develop new trial designs that allow efficacy demonstration of a new therapy when compared to the current standards of care.

## Inclusion of vulnerable populations in trials

Currently, most clinical trials enroll fit patients with high health literacy and access to academic centers. Accordingly, including frail patients with co-morbidities and/or decreased access to modern clinical care in trials for relapsed/refractory disease is essential. Racial, ethnic and SES diversity of the patient population should be increased in all future trials. Recent review of treatment outcomes of black and white myeloma patients receiving care through the Veterans Administration Healthcare System showed better outcomes for black patients, underscoring that access to health care is key [91]. This will provide access to therapies for otherwise marginalized populations and insights into disease biology and outcomes in these specific subsets. Another group of MM patients excluded from trial participation are those with borderline hematopoietic or renal function. Many of these patients have an excellent performance status but maintain a platelet count below 50–75,000 or a GFR < 30 ml/min and are therefore excluded from studies. Additionally, patients with plasma cell leukemia and non-secretory MM are usually excluded from clinical trials, as are those

with a discordance between disease burden and M protein levels. As patients with relapsed myeloma continue to live longer and seek therapeutic options, understanding the optimal treatments available is increasingly important, especially in the context of translating phase 3 findings to real world practice [90].

The NCI myeloma Steering Committee is committed to addressing these gaps in future trial design. Rapid advances in therapy for multiple myeloma permit consideration of such critical issues as best combinations, appropriate sequencing, and outcomes in specific patient populations.
